# The Role of TARE for Bridging and Downstaging of HCC Before Resection or Liver Transplant

**DOI:** 10.3390/cancers18020225

**Published:** 2026-01-11

**Authors:** Abdullah Alshamrani, Sung Ki Cho, Namkee Oh, Jinsoo Rhu, Gyu-Seong Choi, Dong-Ho Hyun, Jongman Kim

**Affiliations:** 1 Department of Surgery, Samsung Medical Center, Sungkyunkwan University School of Medicine, Seoul 06351, Republic of Korea; abdullah.alshamrani@samsung.com (A.A.); namkee.oh@samsung.com (N.O.); jinsoo.rhu@samsung.com (J.R.); gyuseong.choi@samsung.com (G.-S.C.); 2 Department of Radiology and Center for Imaging Science, Samsung Medical Center, Sungkyunkwan University School of Medicine, Seoul 06351, Republic of Korea; sungkismc.cho@samsung.com

**Keywords:** hepatocellular carcinoma (HCC), living-donor liver transplant (LDLT), surgical resection, transarterial radioembolization (TARE)

## Abstract

**Background**: Hepatocellular carcinoma (HCC) remains a major cause of cancer-related mortality. Transarterial radioembolization (TARE) has emerged as a locoregional therapy to downstage tumors and expand surgical eligibility. **Methods**: This retrospective study included patients with HCC who underwent TARE as a bridging treatment. The primary outcomes assessed were the efficacy of TARE in facilitating curative surgery and long-term oncological outcomes, specifically overall survival (OS) and disease-free survival (DFS). **Results**: This study included 25 patients. 17 patients subsequently underwent surgical resection and eight underwent living-donor liver transplantation (LDLT). At a median follow-up of 33.4 months, the median disease-free survival (DFS) was 11.2 months. Patients with recurrence had a median DFS of 3.65 months, and those without recurrence had a median DFS of 27.1 months. The median overall survival (OS) for the cohort was 33.4 months. At the last follow-up, 76% of patients were alive and disease-free. Kaplan–Meier analysis demonstrated sustained OS in the LDLT group, while resection patients gradually declined within the first two years. **Conclusions**: TARE is an effective bridging strategy that enables curative-intent surgery in selected patients with HCC and supports favorable long-term oncological outcomes. Careful patient selection and multidisciplinary management remain essential to optimize survival benefits.

## Simple Summary

This paper evaluated transarterial radioembolization (TARE) as bridging/downstaging therapy followed by surgical resection or liver transplantation in patients with hepatocellular carcinoma (HCC). Yttrium-90 TARE was administered to 25 patients, and liver resection was performed on 17 patients and liver transplantation by living donors on 8 patients. TARE was efficient in inducing tumor necrosis and stimulating growth of the subsequent liver remnant, thus permitting surgery in patients who were initially not suitable to receive a definitive treatment. By the final follow-up, 76% of the patients were alive and disease free, and few patients were found to have early recurrence. This evidence indicates that TARE is a potentially useful preoperative strategy in increasing curative treatment in patients with HCC.

## 1. Introduction

Patients suffering from hepatocellular carcinoma (HCC) are often diagnosed in an advanced stage of the disease, which makes them ineligible for immediate life-saving therapies like surgical removal of the tumor or liver transplantation [1,2]. Transarterial radioembolization (TARE) using Yttrium-90 (Y-90) is now one of the most aggressive local therapies for controlling tumors and facilitating right or left hemiliver hypertrophy. Thus, it links to curative surgery in formerly unresectable patients [3]. Initially, TARE was used only for palliative purposes, but increasing evidence supports its use for downstaging and bridging purposes before transplantation or resection [4]. As with portal vein embolization, TARE also causes selective radiation-induced atrophy of the treated hemiliver and atrophy and hypertrophy of the contralateral future liver remnant (FLR), but with additional tumoricidal effects [5].

In a landmark review of Korean experience, Kim et al. (2017) described the safety and efficacy of TARE in unresectable HCC patients, reporting longer time to progression, better tumor downsizing, less post-embolization syndrome, and shorter hospitalization compared with conventional transarterial chemoembolization (TACE) [6]. Similarly, Shehta and colleagues reported that in five patients, tumors shrank by approximately 24.5%, and the future liver remnant increased from about 354.6 mL to 500.8 mL, which enabled all patients to undergo resection without major radiation-related complications and with favorable short-term outcomes [7]. These studies support the dual action of TARE in controlling tumor progression and inducing contralateral hypertrophy.

Tabone et al. (2019) reported that in a cohort of 24 patients with unresectable HCC, ~20% were successfully downstaged to resection following Y-90 resin microsphere radioembolization; higher tumor-absorbed radiation doses and lower baseline AFP levels correlated with successful downstaging [8]. Moreover, a multidisciplinary working group recently provided recommendations on converting previously unresectable HCC to resection status after radioembolization, emphasizing optimal patient selection, timing, and imaging follow-up strategies to maximize safety and efficacy during the bridging window [9]. Despite the promising data, significant gaps remain. Many series are retrospective and limited in sample size, especially for resection-conversion settings; the optimal radiation dose thresholds or biomarkers predictive of response are not clearly defined; comparative prospective trials between TARE and other loco-regional strategies (TACE, ablation) in bridging or downstaging contexts are scarce; and long-term oncological outcomes (beyond short- or mid-term follow-up) in the resection or transplant-context remain underexplored. Therefore, this study aims to evaluate the efficacy and safety of TARE in patients with HCC.

## 2. Methodology

### 2.1. Patients

This retrospective observational study included patients with hepatocellular carcinoma (HCC) who underwent TARE within a defined study period at a tertiary liver cancer center. Eligible patients were those diagnosed with HCC based on imaging criteria and who underwent TARE as a bridging or downstaging strategy and subsequently received either surgical resection or living-donor liver transplantation (LDLT). Patients were considered initially unsuitable for curative surgery due to tumor size, multifocal disease, vascular involvement, or inadequate future liver remnant (FLR). Patients who received additional locoregional treatments (e.g., TACE or RFA) in combination with TARE were included if TARE was the main bridging or downstaging modality. Patients with extrahepatic spread, advanced hepatic decompensation (Child–Pugh C), uncontrolled comorbidities, or other locoregional therapies (e.g., TACE or RFA alone) were excluded. Patients lacking adequate pre- or post-TARE imaging or clinical follow-up data were also excluded.

### 2.2. Data Collection

Clinical and oncological data were extracted from medical records. Baseline parameters included patient demographics, liver function tests, tumor burden, and imaging findings. TARE treatment details were collected, such as microsphere dose, dosimetry, and distribution. HCC patients were reassessed for surgical eligibility using imaging, tumor markers including alpha-fetoprotein (AFP) and protein induced by vitamin K absence or antagonist-II (PIVKA-II), and liver function after TARE. Surgical outcomes included type of surgery (resection and LDLT), operative parameters (margin status, blood loss, complications), and recovery (hospitalization and liver function recovery). Oncological outcomes assessed recurrence-free survival (RFS) and overall survival (OS).

### 2.3. TARE Procedure

All patients underwent hepatic angiography and mapping before Y-90 administration to assess vascular anatomy and minimize non-target embolization. The dose was individualized according to tumor size, liver volume, and lung shunt fraction. Following treatment, patients underwent regular clinical and radiological follow-up to evaluate tumor response, hypertrophy of the future liver remnant, and liver function. Conversion surgery was considered if adequate FLR was achieved and extrahepatic disease remained absent. Resection was performed in patients with preserved hepatic reserve, while LDLT was chosen for those with cirrhosis or insufficient FLR.

The tumor-to-normal liver (T/N) ratio was assessed using post-TARE imaging by comparing the degree of radiotracer uptake within the treated tumor to that of the adjacent non-tumorous liver parenchyma on Y-90 PET/CT or bremsstrahlung SPECT imaging. Regions of interest were manually defined over the tumor and surrounding normal liver, and the T/N ratio was calculated as the relative uptake intensity of tumor tissue divided by normal liver tissue.

Tumor radiation dose was estimated using post-treatment dosimetry based on delivered Y-90 activity and tumor volume. Absorbed dose calculations were derived from standard dosimetric models using Y-90 PET/CT or bremsstrahlung SPECT imaging. Tumor dose was calculated as the absorbed radiation dose to the tumor tissue expressed in Gray (Gy).

Bridging was defined as the use of TARE to control tumor progression in patients who were already within accepted criteria for surgical resection or liver transplantation while awaiting definitive surgery. Downstaging was defined as the use of TARE to reduce tumor burden in patients who initially exceeded surgical or transplant criteria, enabling subsequent eligibility for curative resection or LDLT.

Tumor dosimetry data were unavailable in 7 patients because post-treatment quantitative imaging suitable for dosimetric analysis was not routinely performed in the earlier part of the study period or was technically inadequate for reliable measurement. These patients were therefore included in clinical and oncologic outcome analyses but excluded from dosimetry-specific reporting.

### 2.4. Statistical Analysis

All analyses were performed using the SPSS software (version 29.0), developed by SPSS Inc., based in Chicago, IL, USA, for the statistical analyses. Continuous variables were expressed as median (range) and compared using Mann–Whitney U tests. Categorical variables were compared using Chi-square or Fisher’s exact tests as appropriate. Survival outcomes (RFS and OS) were estimated using Kaplan–Meier methods, and differences between groups were analyzed with the log-rank test. A *p*-value < 0.05 was considered statistically significant.

The different surgical procedures were analyzed together because the primary objective of this study was to determine the role of TARE as a bridging or downstaging strategy enabling curative-intent surgery, regardless of the specific operative technique. All procedures aimed at complete tumor removal or replacement of the diseased liver, and outcomes were assessed using shared oncologic endpoints, including disease-free survival and overall survival.

The study followed institutional ethical guidelines and received approval from the hospital’s Institutional Review Board Samsung Medical center (SMC-2025-10-110). We omitted the requirement for informed consent due to the retrospective nature of this analysis of de-identified data.

## 3. Result

### 3.1. Baseline Characteristics

A total of 25 patients diagnosed with hepatocellular carcinoma (HCC) were included in this study, comprising 17 males (68%) and 8 females (32%), indicating a male predominance. The mean age was 57.76 years for males and 58.63 years for females. The mean body mass index (BMI) for the cohort was 24.36 kg/m^2^, ranging from 16.11 to 32.10 kg/m^2^, with male patients averaging 24.10 kg/m^2^ and female patients 24.98 kg/m^2^; notably, one patient was classified as obese with a BMI ≥ 30 kg/m^2^. Table 1 gives a detailed demographic overview. Fifteen patients (60%) had HCC, while 10 patients (40%) presented with hepatocellular carcinoma or necrotic nodules, with no significant gender differences in diagnosis. Baseline laboratory parameters revealed comparable hematological profiles between patients undergoing surgical resection and living-donor liver transplantation (LDLT). However, the LDLT group exhibited higher bilirubin levels and prolonged INR, reflecting more advanced liver dysfunction. Baseline tumor markers, including AFP and PIVKA-II, were elevated in the surgical resection group, whereas inflammatory markers such as CRP showed no significant differences. Liver stiffness assessments indicated that 50% of LDLT patients had advanced fibrosis (F4, ≥11 kPa), 25% had moderate fibrosis (F3, 8.1–10.9 kPa), and 25% had mild fibrosis (F2, <8 kPa). CAP values ranged from 166 to 301 dB/m, and increased spleen stiffness corresponded with higher fibrosis stages, highlighting a predominance of moderate-to-advanced liver damage within the cohort. Table 1 presents baseline demographic, clinical, and pathological characteristics of patients.

### 3.2. Pre-Operative Factors

Of the 25 patients, 17 underwent surgical resection and 8 underwent LDLT. The most common surgical intervention was right hemihepatectomy (9 cases, 37.5%), followed by LDLT (8 cases, 32%), laparoscopic extended right hemihepatectomy (3 cases, 12.5%), and segmentectomy (2 cases, 8.3%). Less frequently performed procedures included caudate lobectomy, laparoscopic S2/S3 wedge resection, laparoscopic extended left hemihepatectomy, and laparoscopic central hepatectomy involving segments 4, 5, and 8. Secondary procedures were occasionally required, with cholecystectomy being most common (2 cases, 8.3%), and single instances of open cholecystectomy, abdominal adhesiolysis, and en bloc diaphragm resection. LDLT procedures were associated with significantly longer operation times (*p* < 0.001) and higher estimated blood loss (*p* = 0.011) compared to surgical resection, reflecting increased procedural complexity. ASA classification showed a higher proportion of ASA 3 patients in the LDLT group, indicating greater preoperative risk. Age distribution revealed that older patients predominantly underwent major hepatic resections such as right or left hemihepatectomies, while LDLT was often performed in younger candidates with preserved hepatic function.

### 3.3. Pathology

Pathological analysis included 18 HCC cases and 7 post-treatment necrotic nodules. Tumor sizes varied widely, ranging from 1.2 cm to 12.5 cm, and one patient exhibited more than 30 tumors across multiple hepatic segments (S2, S4, S7, S8), demonstrating extensive intrahepatic spread. The most frequently involved segments were S5, S6, S7, and S8, with multinodular cases such as five in S8 and 14 in S7. Cirrhosis or incomplete cirrhosis was present in approximately half of the cohort. Microvascular invasion was observed in several cases, although portal vein invasion was absent. Surgical margins were negative in all patients, ranging from 0.1 to 4 cm. Solitary tumors were more common in the surgical resection group. In contrast, LDLT patients exhibited higher rates of microvascular invasion (*p* = 0.028) and intrahepatic metastasis (*p* = 0.010). Necrotic nodules consistently demonstrated 100% necrosis, while HCC cases displayed variable necrosis ranging from 1% to 99.5%, reflecting the heterogeneity of treatment response (Table 2).

### 3.4. TARE

Transarterial radioembolization (TARE) and transarterial chemoembolization (TACE) were widely used as preoperative interventions. Multiple HCC was the primary indication, observed in 4 surgical resection patients (23.5%) and all 8 LDLT patients (100%). Other indications, including portal vein tumor thrombosis with satellite nodules (2 cases, 11.8%) and small remnant liver volume (3 cases, 17.6%), were exclusive to the surgical resection group. Table 3 categorizes tumors based on the degree of necrosis observed post-TARE, alongside the corresponding tumor-to-normal liver (T/N) ratios, radiation doses delivered, and procedure-related complications. TARE outcomes were variable; while some patients achieved complete devascularization, others experienced persistent or recurrent lesions. Adverse events included renal dysfunction with myalgia, liver function test elevation with fever, nausea, vomiting, generalized abdominal discomfort, and radiation pneumonia. Complications were more common in patients with cirrhosis, whereas incomplete cirrhosis or septal fibrosis was associated with a broader range of side effects. Tumor progression was observed in patients treated with TACE alone, whereas patients who received combined TACE and TARE showed stable disease during the observation period. The interval between TARE and surgery ranged from 5.6 to 34 months, with a median of 9.1 months, reflecting individualized clinical decision-making. Key procedural outcomes across all cases were evaluated to analyze the efficacy and safety of transarterial radioembolization (TARE) in hepatocellular carcinoma. The detailed clinical, laboratory, imaging, and treatment characteristics of patients undergoing transarterial radioembolization (TARE) are presented in Table 4. In Table 4, tumor radiation dose is reported as two values which show the range of absorbed radiation doses measured across different tumor regions or treated segments.

### 3.5. Outcomes

**Figure 1:**
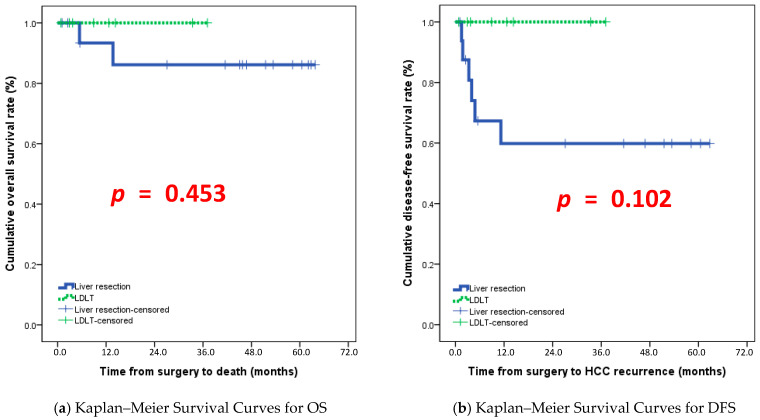
Kaplan–Meier survival curves comparing liver resection and living-donor liver transplantation (LDLT).

During follow-up, 6 patients (24%) experienced disease recurrence, and 2 patients (8%) died, both following recurrences. The median disease-free survival (DFS) for the entire cohort was 11.2 months. Among patients who developed recurrence, the median time to recurrence was 3.65 months, whereas patients without recurrence remained disease-free for a median follow-up of 27.1 months. Median overall survival (OS) was 33.4 months overall; paradoxically, patients with recurrence had higher median OS (45.45 months) than those without recurrence (27.1 months), likely due to follow-up timing differences, shown in Figure 1. Surgical resection patients had significantly longer follow-up (*p* = 0.030), suggesting better long-term outcomes, whereas LDLT patients with more advanced disease had more complex surgeries and higher intraoperative risk. Multimodal treatment strategies combining TARE, TACE, RFA, chemotherapy, and radiotherapy were integral in optimizing tumor control, downstaging disease, and improving resectability. Liver stiffness and CAP assessments highlighted that the majority of patients had moderate-to-advanced fibrosis, influencing both treatment tolerance and post-procedural recovery. Postoperative care emphasized monitoring for radiation-induced liver injury, portal vein thrombosis, and hepatic dysfunction to ensure optimal recovery. Kaplan–Meier survival analysis demonstrated a gradual decline in survival in the surgical resection group during the first two years. In contrast, LDLT patients maintained nearly 100% survival throughout the observed period, illustrating the complexity of patient outcomes and the impact of individualized treatment strategies.

Postoperative complications were classified according to the Clavien–Dindo system. Most complications were minor (Clavien–Dindo grade I–II) and included transient liver enzyme elevation, nausea, vomiting, and fever, which were managed conservatively. A limited number of patients experienced major complications (grade III or higher), requiring interventional or intensive management. No perioperative mortality (grade V) was observed.

## 4. Discussion

Postoperative complications and the requirement for intensive care unit (ICU) support reinforce the need for comprehensive post-surgical management. The presence of radiation-induced liver injury, portal vein thrombosis, and treatment-related hepatic dysfunction in some patients underscores the complexity of post-treatment recovery.

### 4.1. Surgical Management of HCC

This study provides meaningful insights into the surgical management of hepatocellular carcinoma (HCC), highlighting key patterns in treatment approaches, surgical outcomes, and postoperative complications. Right hemihepatectomy was the most frequently performed surgical procedure in this cohort, reflecting its continued use in selected patients with anatomically suitable disease and adequate liver reserve [10,11]. Consistent with earlier research, our findings suggest that resection continues to be widely adopted where donor availability is limited, despite the associated risks [12,13]. Importantly, living-donor liver transplantation (LDLT) was associated with fewer postoperative complications compared with resection, aligning with previous studies that underscore the protective role of transplantation in patients with cirrhosis and compromised hepatic reserve [10,11,14,15,16,17]. These findings reinforce LDLT as a compelling alternative, particularly in well-selected younger patients with preserved functional capacity and access to suitable donors.

### 4.2. TARE Response and Tumor Control

Preoperative interventions played a decisive role in shaping surgical feasibility. In particular, transarterial radioembolization (TARE) was instrumental in tumor downstaging and improving resectability [2,18]. The presence of necrotic nodules in post-treatment pathological evaluations further validates the therapeutic potential of locoregional therapies [19]. Multiple studies have suggested the feasibility of resection following TARE. Studies reported that 27.7% of patients with single large HCC (≥5 cm) treated with TARE could undergo subsequent resection, achieving comparable overall survival and postoperative outcomes to upfront surgical candidates, especially when higher radiation doses were administered (mean ^90^Y dose 211.89 Gy vs. 128.7 Gy, *p* < 0.001) [20,21]. Similarly, another study suggested that radiation lobectomy using TARE significantly increased FLR volume over 9 months and correlated with reduced gadolinium-EOB uptake on MRI, marking functional parenchymal changes predictive of resectability [3,9]. Consensus guidelines and expert working groups have now recommended using TARE for conversion to resection in carefully selected patients, emphasizing its dual role in achieving hypertrophy and oncologic control [9].

In addition to favorable clinical outcomes, TARE has shown advantages in cost-effectiveness and bridging success. Wu et al. found TARE more cost-effective than TACE for downstaging in liver transplant candidates, yielding more quality-adjusted life years (QALYs) with only marginally higher costs [22]. A pooled analysis study affirmed that TARE produces higher downstaging rates than TACE and supports its application in patients beyond the Milan criteria. However, indications for its use in transplant settings remain variable [4]. Furthermore, Korean institutional experiences have highlighted the utility of TARE in inducing tumor necrosis and vascular remodeling while maintaining acceptable safety profiles, particularly in the context of radiation lobectomy protocols [3].

Recent studies support the expanding role of TARE in HCC beyond traditional palliation. Multiple cohorts have shown that TARE can maintain transplant eligibility and achieve successful downstaging in patients whose tumors initially exceed transplant criteria, with up to ~30% of patients undergoing liver transplantation after TARE downstaging in larger series [23]. Evidence also indicates that TARE may act as a bridge to surgery for patients awaiting transplantation, maintaining tumor stability and allowing time for definitive therapy [24].

However, this benefit must be weighed against the risk of adverse events, including hepatic function derangements and renal impairment, which were observed in our cohort. These complications mirror those reported in larger series, where the tolerability of TARE was closely linked to baseline liver function and degree of fibrosis [25,26]. Careful patient selection and vigilant post-procedural monitoring remain critical to balance efficacy with safety.

When stratified by age, younger patients were more likely to undergo LDLT, whereas older patients tended to receive resection. This pattern reflects surgical eligibility, donor availability, and liver reserve, consistently with prior reports [15,27]. Our findings also highlight that advanced fibrosis and cirrhosis influence treatment allocation and postoperative outcomes, with such patients experiencing more TARE-related adverse effects and surgical complications, in line with previous studies [11,25].

Recent studies have introduced the concept of borderline resectable HCC to better identify patients who can benefit from neoadjuvant or bridging therapies such as TARE before definitive surgery. One international validation refined borderline resectability criteria by integrating the Tumor Burden Score and other clinical factors, categorizing patients into resectable, BR1, and BR2 groups with distinct survival outcomes after surgery [28]. Expert consensus from the Japan Liver Cancer Association and the Japanese Society of Hepato-Biliary-Pancreatic Surgery defines oncological resectability categories (R, BR1, BR2) based on tumor size, number, and invasion patterns to guide multidisciplinary treatment decisions [29].

### 4.3. Prognostic Role of Recurrence

The study also sheds light on the prognostic role of recurrence. Early recurrence, particularly within the first-year post-surgery, was strongly associated with significantly worse overall survival (OS). This finding parallel reports from other series emphasizing the aggressive biology of early-relapsing HCC and its detrimental impact on long-term outcomes [30,31]. Yet, observing that some patients with recurrence achieved durable survival highlights the importance of post-recurrence management strategies, such as repeat resection, salvage transplantation, or locoregional therapies. When tailored to the individual, these options can substantially modify the survival trajectory, as noted in recent comparative studies [32,33]. Interestingly, our cohort’s variable interval between TARE and surgery did not independently predict survival outcomes. This suggests that timing alone is not the decisive prognostic factor but a constellation of clinical, pathological, and technical variables that interact to shape prognosis.

### 4.4. Comparison of Surgical Resection and LDLT Outcomes

Comparison with prior literature underscores several converging and diverging trends. While our study observed higher rates of complications following surgical resection, including nausea, vomiting, liver enzyme derangements, and radiation pneumonia, these are in line with existing evidence that major hepatic resections carry increased perioperative risks [34]. However, margin negativity was more commonly achieved in resection cases than in LDLT. This suggests that although transplantation may be protective against complications and recurrence in cirrhotic patients, aggressive resection still plays an irreplaceable role in curative intent, particularly when anatomical considerations favor clear margin achievement [35]. Another important observation was the association between microvascular invasion, multicentric disease, and recurrence. This reiterates findings from prior studies that microscopic tumor spread remains a key challenge in achieving durable cures. As suggested in previous reports, strategies such as intraoperative ultrasound, frozen section analysis, and expanded resection margins may help mitigate these risks and improve long-term disease control [36].

### 4.5. Long-Term Outcomes

These findings emphasize that no single modality offers a universal solution. LDLT, resection, and locoregional therapies all contribute distinct benefits and risks, and optimal outcomes depend on integrating them into an individualized, multidisciplinary treatment framework. For patients with preserved hepatic function and favorable anatomy, resection offers curative potential with the advantage of immediate availability. For those with cirrhosis or advanced fibrosis, LDLT provides a safer postoperative profile and improved long-term outcomes but is constrained by donor limitations. Meanwhile, when judiciously applied, TARE represents a valuable adjunct in multimodal planning, enhancing resectability and tumor control.

### 4.6. Limitations

Despite its contributions, this study has important limitations. The relatively small sample size (n = 25) restricts statistical power and limits generalizability. Larger multicenter cohorts are needed to support these trends. The retrospective design also introduces potential biases in reporting complications and outcomes, and patient-reported measures were not systematically captured. Another limitation is the lack of extended follow-up data to fully evaluate long-term survival and recurrence patterns, both of which are crucial indicators of treatment efficacy. The variability in preoperative treatments, particularly in the type and timing of locoregional therapies, complicates efforts to isolate the independent effect of each intervention. Compared with previous studies that focused on homogeneous patient populations or a single curative pathway [4,37], our cohort included both resection and LDLT patients with heterogeneous tumor burden, which may limit direct comparison with previously reported outcomes.

## 5. Conclusions

This study emphasizes the evolving landscape of liver cancer treatment, where multimodal strategies, including preoperative locoregional therapies and surgical interventions, are essential for optimizing patient outcomes. LDLT demonstrated a lower complication rate than surgical resection, highlighting its value in selected patient populations. The effectiveness of TARE in tumor downstaging was evident, though individual responses varied, reinforcing the need for patient-specific treatment planning. Importantly, patients were observed for approximately six months after TARE, allowing for a meaningful assessment of treatment response and its impact on surgical resectability and postoperative outcomes.

## Figures and Tables

**Table 1:** Baseline Demographic Data.

Parameters	Surgical Resection	LDLT	*p*-Value
	(n = 17)	(n = 8)	
Sex (Male)	16 (94.1%)	6 (75.0%)	0.231
Age (Years)	61 (37–78)	57 (41–71)	0.673
BMI (kg/m^2^)	25.3 (19.5–32.1)	22.3 (16.1–29.8)	0.673
WBC (×10^9^/L)	5020 (1930–13,100)	5045 (1710–13,010)	1.000
NLR	0.40 (0.09–1.01)	0.31 (0.06–0.59)	0.673
Hemoglobin (g/dL)	13.5 (9.7–15.9)	12.1 (6.5–17.0)	1.000
Total bilirubin (mg/dL)	0.6 (0.2–1.4)	2.9 (0.3–20.8)	0.081
AST (U/L)	27 (15–62)	33 (18–300)	0.389
ALT (U/L)	20 (9–69)	26 (12–140)	0.411
INR	1.01 (0.89–1.12)	1.16 (0.96–1.45)	0.010
Creatinine (mg/dL)	0.78 (0.61–1.68)	0.74 (0.50–2.37)	0.673
CRP (mg/L)	0.47 (0.09–6.85)	0.19 (0.03–6.76)	0.637
Time from TARE to surgery (months)	8.9 (5.6–34.0)	10.7 (5.8–29.2)	0.411
Radiation lobectomy	5 (29.4%)	0 (0%)	0.140
Scheduled surgery	8 (47.1%)	2 (25.0%)	0.402
Initial AFP (ng/mL)	7.8 (1.3–2751)	3.6 (2.4–21.0)	0.042
Initial PIVKA-II (mAU/mL)	941 (19–49,984)	15 (9–189)	0.002
Preoperative AFP (ng/mL)	4.3 (2.4–149.3)	3.7 (1.6–145.0)	0.234
Preoperative PIVKA-II (mAU/mL)	40 (14–486)	50 (18–28,293)	1.000
ASA			<0.001
1	0	1(12.5%)	
2	17(100%)	2(25%)	
3	0	5(62.5%)	

**Table 2:** Tumor Pathology Features.

Tumor Pathology Features			
Parameters	Surgical Resection	LDLT	*p*-Value
	(n = 17)	(n = 8)	
Maximum tumor size (cm)	4.2 (1.5–12.5)	4.5 (1.0–16.2)	1.000
Tumor number (solitary)	14 (82.4%)	2 (25.0%)	0.012
Tumor grade III	1 (9.1%)	0 (0%)	0.539
Microvascular invasion	4 (23.5%)	6 (75.0%)	0.028
Serosal involvement	1 (5.9%)	2 (25.0%)	0.231
Intrahepatic metastasis	3 (17.6%)	6 (75.0%)	0.010
Tumor necrosis			
Total	9 (52.9%)	5 (62.5%)	
50–99%	7 (41.2%)	1 (12.5%)	0.171
<50%	1 (5.9%)	2 (25.0%)	
Follow-up duration (months)	45.8 (1.1–63.8)	10.8 (0.8–37.1)	0.030

**Table 3:** Tumor Necrosis (T/N) Summary of Transarterial Radioembolization.

Tumor Necrosis Category (T/N)	Number of Cases (n)	Mean T/N Ratio	Radiation Dose (Gy)	Complications Observed
Near-complete/Complete	14	7.8	462 ± 150	None reported; mild LFT elevation in a few cases, radiation pneumonia
Partial	8	4.9	270 ± 120	Mild abdominal pain in 2 cases; otherwise none
Minimal/Poor	3	2.5	122 ± 50	None reported

**Table 4:** Clinical characteristics, treatment details, and outcomes of patients who underwent transarterial radioembolization (TARE).

Pt. No	TARE Reason	TARE Side Effects	Procedure	Initial		Pre-Op		Y-90 Dose (GBq)	Tumor Radiation Dose (Gy)	TTS	TTR
				AFP	PIVKA-II	AFP	PIVKA-II				
1	Abdominal aortic aneurysm	Renal dysfunction, myalgia	TARE	1.3	13,235	8.1	394	Multiple infusions	N/A	7.1	10.3
2	Satellite nodules	none	TARE	17.3	193	149.3	25	N/A	N/A	10.8	12.2
3	Satellite nodules, small remnant liver volume	LFT elevation, Fever	TARE	5.5	30,324	4.3	20	Resin-type	N/A	6.1	68.8
4	Satellite nodule	none	TACE + TARE	50.4	49,984	2.6	113	3.7	170	8	12
5	small remnant volume	nausea, vomiting, poor oral intake	Selective TARE	1.3	105	2.4	32	36	N/A	6.3	66.7
6	Satellite nodules, small remnant liver volume, stomach cancer	nausea, vomiting, pain	TARE	2751	941	3.8	33	2.8	413.69	10.0	68.1
7	small remnant volume	Generalized abdominal discomfort	TARE + cTACE	5.5	1242	6	40	Resin-type	N/A	5.6	58.9
8	PVTT, irregular mass, satellite nodules	none	TARE Segmentectomy	3	1357	3	184	Glass-type (glass Y-90 microspheres)	N/A	6.7	8.5
9	Two HCC	none	Selective TARE	8.6	1460	6.9	31	4.62	344–120	11.5	62.9
10	Satellite nodule	none	Right Lobar TARE	16.3	709	6.8	56	4.42	120–240	7.1	53.8
11	Infiltrative HCC with PVTT	none	TARE	16.9	7231	2.5	63	3 + 10	120–240	34.0	38.8
12	Large HCC, satellite nodules	Radiation pneumonia	TARE	32	1141	4.1	17	3.9	122–211.9	7.4	18.6
13	small remnant volume	none	Right Lobar TARE	1121	297	10.7	44	2.5	269.7	8.9	50.4
14	PCI unstable angina., DM, advanced LC (Plt 69,000)	none	TARE	6.3	706	5.3	14	5	216–233	18.3	45.4
15	Multiple HCC, irregular margin	none	Right Lobar TARE	21	11	145	38	4	354.4	10.8	23.5
16	Multiple HCC	none	TARE Segmentectomy	3.2	12	3.6	61	22	250–240	29.2	66.2
17	Multiple HCC	none	TARE Segmentectomy	2.4	12	1.9	18	2.01	120–240	25.3	58.6
18	Multiple HCC	none	TARE + cTACE	5.1	18	4	37	5.23	420	6.0	20.2
19	Multiple HCC	none	Right Lobar TARE	2.5	162	3.8	1271	5.8	164.03	15.3	18.2
20	Multiple HCC	none	Segmental TARE	7.8	69	17.9	486	3.98	625	14.3	16.8
21	Multiple HCC	none	Segmental TARE	3.5	19	4.1	19	4.18	907–403	15.5	16.6
22	Multiple HCC	none	Right Lobar TARE	5.1	17	12.6	68	5.01	462	5.8	14.7
23	Multiple HCC	none	TARE Segmentectomy	2.2	26	4.1	44	3.98	603	9.0	14.5
24	Multiple HCC	none	TARE Segmentectomy	3.9	9	1.6	24	7.95	618	10.6	14.3
25	Multiple HCC	none	TARE	3.2	189	1.6	28293	N/A	N/A	9.1	9.9

## Data Availability

The data supporting this study’s findings are available from the corresponding author upon reasonable request. Due to patient privacy concerns, the data are not publicly shared.

## Abbreviations

HCC	Hepatocellular Carcinoma
TARE	Transarterial Radioembolization
LDLT	Living-Donor Liver Transplantation
TACE	Transarterial Chemoembolization
RFA	Radiofrequency Ablation
FLR	Future Liver Remnant
ASA	American Society of Anesthesiologists
AFP	Alpha-Fetoprotein
PIVKA-II	Protein Induced by Vitamin K Absence-II
CRP	C-Reactive Protein
LFT	Liver Function Test
OS	Overall Survival
DFS	Disease-Free Survival
CAP	Controlled Attenuation Parameter
HBV	Hepatitis B Virus
HCV	Hepatitis C Virus
EBL	Estimated Blood Loss
TTS	Time to Surgery
TTR	Time to Recurrence
